# Development and Validation of a Quantitative Score for the Criteria Clinical Control in Stable COPD Proposed in the Spanish COPD Guidelines (GesEPOC): Results of the EPOCONSUL Audit

**DOI:** 10.3390/jcm14030707

**Published:** 2025-01-22

**Authors:** Myriam Calle Rubio, Juan José Soler Cataluña, Marc Miravitlles, Bernardino Alcázar Navarrete, José Luis López-Campos, Manuel E. Fuentes Ferrer, Juan Luis Rodríguez Hermosa

**Affiliations:** 1 Pulmonology Department, Hospital Clínico San Carlos, 28040 Madrid, Spain; mcallerubio@gmail.com; 2 Department of Medicine, School of Medicine, Universidad Complutense de Madrid, Instituto de Investigación Sanitaria del Hospital Clínico San Carlos (IdISSC), CIBER de Enfermedades Respiratorias (CIBERES), 28003 Madrid, Spain; 3 Pulmonology Department, Hospital Arnau de Vilanova-Lliria, 46160 Valencia, Spain; jjsoler@telefonica.net; 4 Medicine Department, Universitat de València, CIBER de Enfermedades Respiratorias (CIBERES), Instituto de Salud Carlos III, 28003 Madrid, Spain; 5 Pulmonology Department, Hospital Universitari Vall d’Hebron, Vall d’Hebron Institut de Recerca (VHIR), Vall d’Hebron Barcelona Hospital Campus, CIBER de Enfermedades Respiratorias (CIBERES), 08035 Barcelona, Spain; marcm@separ.es; 6 Pulmonology Department, Hospital Virgen de las Nieves, 18071 Granada, Spain; balcazarnavarrete@gmail.com; 7 IBS-Granada, Medicine Department, Universidad de Granada, 18071 Granada, Spain; 8 Respiratory Disease Medical-Surgical Unit, Instituto de Biomedicina de Sevilla (IBiS), Hospital Universitario Virgen del Rocío/Universidad de Sevilla, 41013 Sevilla, Spain; lcampos@separ.es; 9 CIBER de Enfermedades Respiratorias (CIBERES), Instituto de Salud Carlos III, 28003 Madrid, Spain; 10 Research Unit, Hospital Universitario Nuestra Señora de Candelaria, 38010 Santa Cruz de Tenerife, Spain; mfuentesferrer@gmail.com; 11 Preventive Medicine Department, Hospital Universitario Nuestra Señora de Candelaria, 38010 Santa Cruz de Tenerife, Spain

**Keywords:** chronic obstructive pulmonary disease, degree clinical control, predicted probability, score

## Abstract

**Introduction/Objective**: the concept of clinical control of COPD is a measure proposed in the Spanish COPD Guidelines (GesEPOC), which aims to help clinicians assess the clinical status in order to adapt the treatment plan at follow-up. However, studies that have evaluated clinical practice reveal that the degree of control of COPD is not always assessed, which underlines the need to promote its assessment through a scoring system. To develop a scoring system that quantitatively assesses the validated criteria defining the degree of COPD control. **Methods**: this study used data from the EPOCONSUL audit in respiratory clinics across Spain. We included in this analysis all patients with a COPD clinical control grade estimated and reported by the physician at the visit, who had registered the criteria necessary to define the degree of clinical control validated and established in GesEPOC. Patients were randomly assigned to either the development or validation cohorts. The development cohort included 485 patients and the validation cohort included 341 patients. Score modelling was conducted using a multivariate logistic regression model, and calibration of the model and score was assessed using the Hosmer-Lemeshow goodness-of-fit test and GiViTi Calibration belts. The model and generated score’s discrimination capacity were analyzed by calculating the Area Under the Curve (AUC). **Results**: the scoring system was developed using four criteria as predictors of poor clinical control of COPD reported by the treating physician:adjusted dyspnoea severity, use of rescue inhaler more than three times per week, walking less than 30 min per day, and COPD exacerbations in the last three months. The scoring system attributed scores from 0 to 8. Calibration was satisfactory in both development and validation cohorts, and the score’s discrimination power, as indicated by the AUC, was 0.892. **Conclusions**: this scoring system provides an easy-to-use quantitative assessment of clinical control of COPD that we believe will help to measure COPD control and its evolution during patient follow-up. Future research will be needed to prospectively evaluate this score as a predictor of outcome.

## 1. Introduction

Chronic Obstructive Pulmonary Disease (COPD) is characterised by frequent exacerbations, leading to increased morbidity and mortality. Clinical practice guidelines for COPD focus on symptom reduction and risk minimisation as the main therapeutic goals [1,2]. Achieving clinical control of the disease is the overall objective in the therapeutic approach, requiring adaptation of the therapeutic plan to the patient’s evolution.

The concept of COPD control is an evaluative element proposed in the Spanish COPD Guidelines (GesEPOC), aimed at aiding clinicians in assessing the clinical status of COPD patients during visits [1]. It is a measure based on two components: clinical impact (degree of dyspnoea, use of rescue medication, limitations in daily physical activity, habitual sputum colour) and stability (absence of exacerbations in the last 3 months) [1,3] that is recommended to be assessed at each visit, similarly to the GOLD guidelines that follow-up treatment be based on two key treatable traits: dyspnoea and occurrence of exacerbations [2]. Previous studies have evaluated the relevance of the variables, thresholds and number of criteria needed to define the clinical control proposed by GesEPOC [4,5,6]. The combination of these variables indicates that the patient has good clinical control when the patient has stability (no exacerbations in the previous 3 months) and a low impact level, defined by a low level of dyspnoea, no expectoration or mucous expectoration, infrequent use of rescue medication and an adequate level of physical activity [4]. COPD control status predicts future exacerbations, as well as providing relevant information on health status and survival prognosis [7,8,9,10].

Therefore, measurement of the degree of COPD control is a key tool for clinicians in the management of COPD patients, as it can avoid both unwarranted nihilism and excessive diagnostic-therapeutic intervention. Despite the scientific evidence supporting the assessment of COPD control in the follow-up of patients, it is rarely practised routinely by clinicians. The 2021 EPOCONSUL audit assessing practice in pulmonology clinics revealed that the extent of clinical management of COPD was only assessed and reported in one quarter of the audited visits [11]. These results underline the need to promote clinician assessment by providing a scoring system that quantifies the validated criteria defining the degree of control, as set out in GesEPOC.

Thus, this study aims to develop a score that quantifies the variables involved in defining the degree of clinical control in COPD, providing a quantitative assessment of clinical control. A scoring system that helps to assess the degree of clinical control of COPD and therefore to make decisions in the follow-up and management of patients with COPD.

## 2. Materials and Methods

The report follows the Transparent Reporting of a multivariable prediction model for Individual Prognosis or Diagnosis (TRIPOD) statement [12] shown in Table S1.

### 2.1. Data Source

This analysis used data from the EPOCONSUL audit. Briefly, the EPOCONSUL audit is an observational, non-interventional, cross-sectional, multi-centre, nationwide study promoted by the Spanish Society of Pneumology and Thoracic Surgery (SEPAR). It is designed to evaluate outpatient care provided to COPD patients in respiratory clinics across Spain based on available medical registry data, with details previously described [11]. The medical staff responsible for the audited practice was not informed of the audit in order to maintain blinding.

Briefly, the recruitment process was intermittent; each month, investigators recruited the clinical records of the first 10 patients diagnosed with COPD who were seen in the outpatient respiratory clinic. These patients were subsequently re-evaluated to determine if they met the inclusion/exclusion criteria described in Table S2. The information collected was historical for clinical data from the last review visit and concurrent for hospital resource information, as described in Table S3. In total, 45 hospitals participated in the 2021 EPOCONSUL audit, and 4225 clinical records of patients treated in outpatient respiratory clinics were evaluated between 15 April 2021, and 31 January 2022. The participating investigators in EPOCONSUL are listed in Table S4.

### 2.2. Participants

We included in this analysis all patients with a COPD clinical control grade estimated and reported by the physician at the visit, who had registered the criteria necessary to define the degree of clinical control validated and established in GesEPOC [1].

### 2.3. Development and Validation Cohorts

The included population was randomly divided, with approximately 60% of the sample used to develop the model (development cohort) and 40% used as a validation cohort to validate and assess the model’s diagnostic capability.

### 2.4. Predictors and Outcome

Based on the evidence and according to the validated criteria defining the degree of clinical control in GesEPOC [4,13,14,15,16], we included the following variables in our model as predictors of poor clinical control of COPD: the degree of dyspnoea according to the modified Medical Research Council (mMRC) scale [15], the use of rescue medication in the last week, the colour of sputum, the degree of self-reported physical activity at the visit, and the absence of exacerbations in the previous three months requiring systemic corticosteroids and/or antibiotics. We categorised these criteria as predictors in our model based on thresholds identified and validated in previous studies [4,5,6] (detailed in Table S5). The outcome of our analysis was poor clinical control of COPD that was estimated and reported at the visit by the treating physician.

### 2.5. Statistical Analysis

Qualitative variables were summarised as frequency distributions and normally distributed quantitative variables were presented as mean and standard deviation (SD), or as median and interquartile range if they did not fit a normal distribution.

In the development cohort, we calculated the crude Odds Ratio (OR) with 95% confidence intervals (CIs) for each of the five criteria necessary to define the degree of clinical control, as validated and established in GesEPOC, for the outcome of poor clinical control of COPD reported by the treating physician. This was done using univariate logistic analysis. Subsequently, the five criteria were included in a multivariate logistic regression model. The selection of the final set of criteria for the score estimation was performed using different strategies, including automatic selection algorithms (backward and forward) and information criteria (Akaike Information Criterion [AIC], Bayesian Information Criterion [BIC]). The final score was created by assigning points to each criterion by dividing each beta coefficient in the model by the lowest beta coefficient, then rounding to the nearest integer or half-integer. The discrimination capacity of the model and generated score was analysed by calculating the Area Under the Receiver Operating Characteristic Curve (AUC) and its 95% CI. Model and score calibration was assessed with the Hosmer-Lemeshow goodness-of-fit test, and GiViTi Calibration belts were also constructed [17]. The red line represents perfect calibration between the predicted probability and observed outcomes. The light and dark grey calibration bands represent the 80% and 95% confidence levels for this predictive model, respectively. If the red line falls within the calibration band, the model fits well when the *p*-value > 0.05.

For validation, the developed score was applied to the validation cohort, and the discrimination and calibration performances were described. Statistical significance was assumed at *p* < 0.05. All analyses were performed using Stata software version 15 (StataCorp LLC, College Station, TX, USA).

## 3. Results

**Figure 1:**
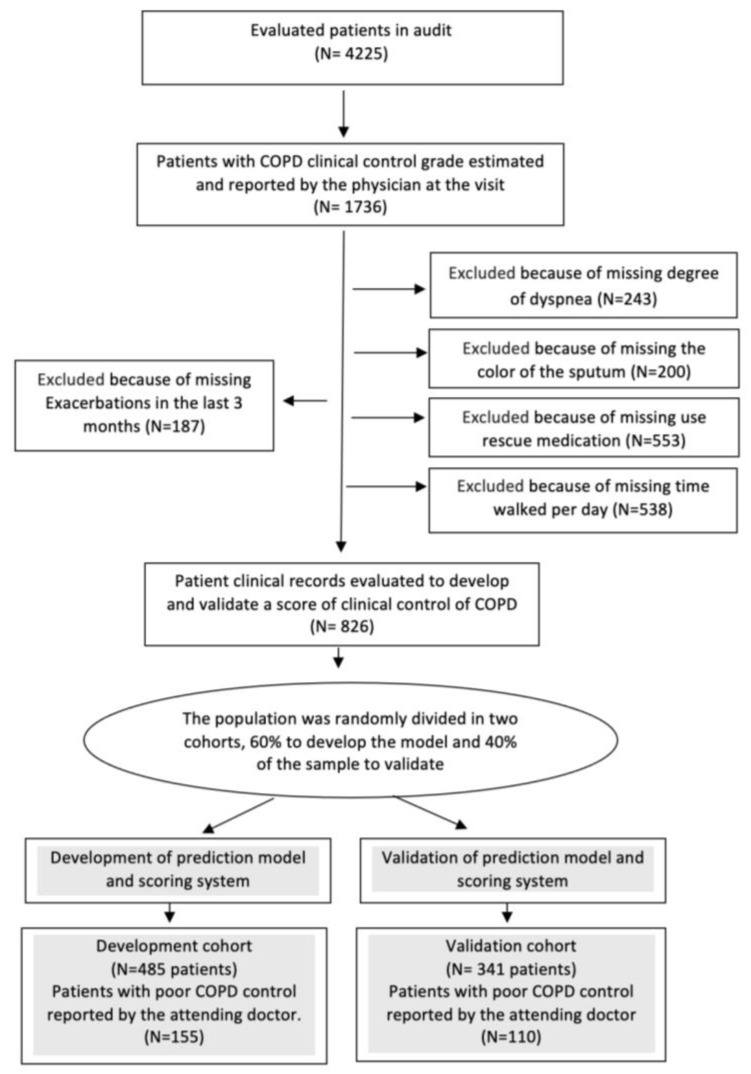
Flow chart of the sampling process.

Among the 4225 patients audited in EPOCONSUL, 826 met the COPD clinical control grade reported at the visit and the criteria to define the degree of clinical control (Figure 1).

A patient may have a missing value in more than one of the criteria.

**Figure 2:**
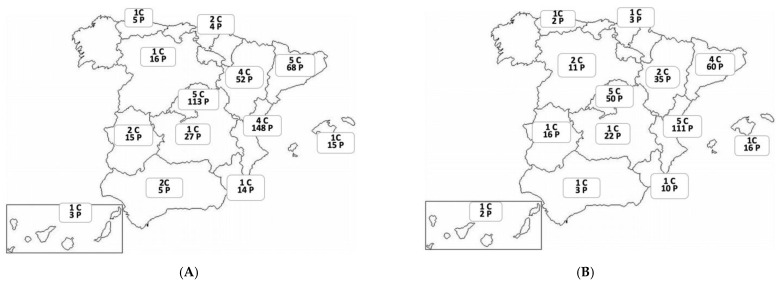
(**A**): Number of participating centres (C) and patients (P) evaluated by regions in development cohort. Data are presented as numbers. (**B**): Number of participating centres and patients evaluated by regions in validation cohort. Data are presented as numbers.

The characteristics of the excluded patients in this analysis are described in Table S6. Figure 2 shows the distribution of centres and patients evaluated by regions in the development and validation cohorts.

The development cohort included 485 patients (mean [SD] age, 68.9 [9.4] years; 341 [70.3%] men) and the validation cohort included 341 patients (mean [SD] age, 69.4 [8.8] years; 240 [70.4%] men). Additional patient characteristics and in-hospital data are described in Table 1. Most participating centers were primarily public university hospitals (development: 337 [69.5%]; validation: 211 [72.8%]). Most patients had moderate to severe obstruction (development: mean [SD] FEV1, 52.7 [17.6%] predicted; validation: mean [SD] FEV1, 52.8 [18.8%] predicted), and nearly half of the patients received inhaled triple therapy (development: 236 [48.8%]; validation: 156 [46.3%]). Poor clinical control of COPD, as reported by the physician at the follow-up visit, was observed in 32% (155) of patients in the development cohort and 32.3% (110) in the validation cohort.

### 3.1. Model Development and Performance

In the development cohort, we conducted an unadjusted analysis of the criteria to define the degree of clinical control in patients with poorly controlled COPD (Table 2). The sputum colour criterion was not retained in the final model to generate the score.

**Figure 3:**
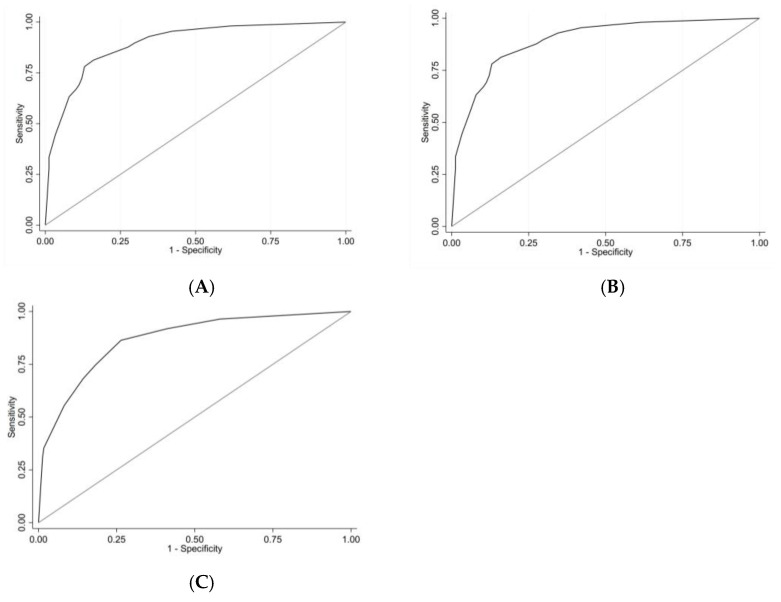
(**A**) Area Under the Receiver Operating Characteristic Curve Modelo Develop. (**B**) Area Under the Receiver Operating Characteristic Curve Score Develop. (**C**) Area Under the Receiver Operating Characteristic Curve Score Validation.

**Figure 4:**
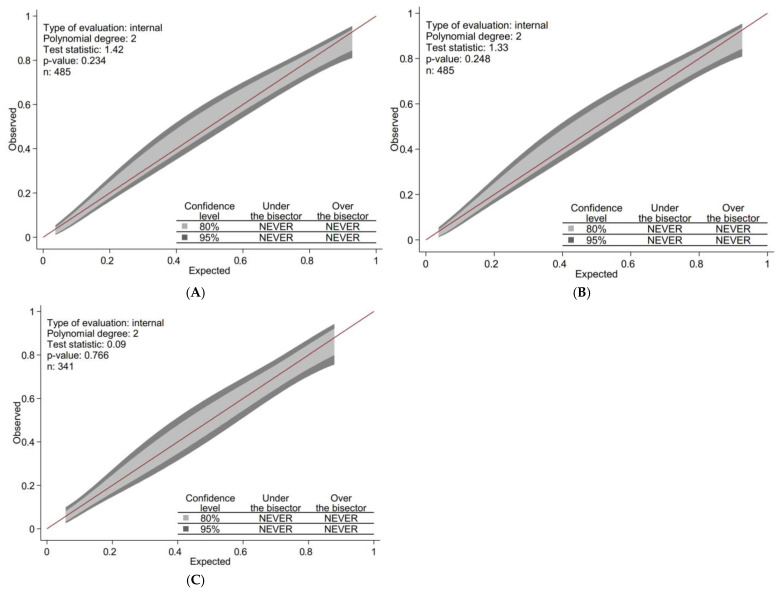
(**A**) Calibraton Belt Modelo Develop. (**B**) Calibraton Belt Score Develop. (**C**) Calibraton Belt Score Validation.

We calculated the β coefficient and OR with 95% CIs using logistic regression analysis (Table 3). The AUC of the model was 0.894 (95% CI, 0.864–0.924, *p* < 0.001) as calculated by the ROC curve (Figure 3A). The Hosmer-Lemeshow test results (χ^2^8 = 2.286, *p* = 0.892) indicated that the prediction was well-calibrated to the observations. Figure 4A shows that the GiViTi calibration curve does not cross the 95% CI area along the 45-degree line (*p* > 0.05).

### 3.2. Score and Grouping

We developed a simple scoring system based on the β coefficient (Table 3). The weight assigned to each criterion is described in Table 3, with a score ranging from 0 to 8, mean (SD) score 2.87 (2.66). The AUC of the score was 0.892 (95% CI, 0.862–0.923, *p* < 0.001) as calculated by the ROC curve (Figure 3B). The Hosmer-Lemeshow test results (χ^2^8 = 4.232, *p* = 0.516) and the calibration belt (Figure 4B) demonstrate good calibration.

### 3.3. External Validation of the Model

In the validation cohort, the AUC of the score was 0.867 (95% CI, 0.826–0.908, *p* < 0.001) (Figure 3C) and the Hosmer-Lemeshow test results (χ^2^8 = 6.007, *p* = 0.306) and the calibration belt (Figure 4C) indicate good calibration.

## 4. Discussion

In this study, we have developed and validated a score that weights the criteria defining the clinical control of COPD. This scoring system aims to provide a quantitative measure of clinical control, promoting its assessment during patient follow-up and facilitating the interpretation of changes over time and their therapeutic implications for clinicians. The score demonstrated good calibration and discrimination accuracy. It includes four criteria defining COPD control according to GesEPOC [1]: dyspnoea, use of rescue medication, impact on physical activity, and exacerbations. These criteria support the premise that COPD control is a multidimensional construct, comprising two evaluative dimensions: a cross-sectional one—clinical impact, or the repercussion of the disease on the patient, and a longitudinal one—stability, defined as the absence of exacerbations [3]. The sputum colour criterion, which was infrequent in our sample, was not associated with poor COPD control and was therefore not retained in the final model. The score demonstrated good calibration and discrimination power.

The assessment of the degree of control in monitoring COPD is a novel element proposed in GesEPOC [1] to aid clinicians in decision-making during visits. However, despite existing guidelines, the assessment of clinical control of COPD is not always evaluated in clinical practice [11], which may lead to a lack of treatment adjustment and COPD patients receiving inadequate treatment with an increased risk of exacerbations and poorer quality of life [18,19,20]. The fact that the degree of COPD control is often not measured or is overestimated by clinicians suggests that current criteria and recommendations alone are insufficient for an adequate assessment of COPD control [21,22]. Therefore, having a scoring system that provides a quantitative measure of the criteria defining clinical control of COPD will help to assess the degree of control during visits. This will also allow measurement of changes in the degree of control over time, as a result of the therapeutic interventions adopted.

The main strengths of the study are its potential relevance and implications in clinical practice as a tool to facilitate the standardization of the assessment of the degree of control in the follow-up of COPD patients. Firstly, the score may assist clinicians in identifying patients with insufficient clinical control of COPD, thereby aiding decision-making during visits. As a more concrete and comparable measure, the score can boost physicians’ confidence in guiding and planning various treatment options, contributing to the appropriate use of medical resources. This is particularly crucial for high-risk populations, allowing for the reassessment of goals, redefinition of necessary therapies [23], and earlier consideration of other associated factors, such as psychosocial issues [24]. Secondly, this quantitative measure of clinical control is easily translatable into routine clinical practice, as it encompasses validated criteria defining the degree of control and pertinent measurements to be conducted during follow-up visits. Previous studies have demonstrated that increased use of rescue medication [25] and low levels of physical activity [26,27] are linked to poorer health outcomes, including a higher risk of future exacerbations, greater deterioration of lung function, and increased all-cause mortality.

In our scoring, the criterion of rescue inhaler use had the highest weight. However, it is a measure not always reported and recorded during clinical practice visits according to the EPOCONSUL audit results, despite its relative ease of assessment [11]. This highlights a crucial point that clinicians often limit their assessment to physiological markers and symptoms [28]. Thirdly, this score provides a means of interpreting changes in the degree of clinical control over time. We anticipate that this score could play a significant role in the assessment of COPD control, similar to the Asthma Control Test (ACT) [29,30] in asthma, and therefore, could be crucial in evaluating therapeutic interventions in COPD in the future. The scoring system translates qualitative criteria into a quantitative and objective tool, allowing clinicians to more accurately identify patients with insufficient control. This facilitates clinical decision-making, treatment adjustments and monitoring of changes over time.

### Study Limitations

This study has several limitations that need to be taken into account when assessing the results. Although this model has been validated in the validation cohort, which makes it potentially applicable to similar settings, our population evaluated consists of COPD patients treated in respiratory clinics, most patients have moderate to severe obstruction, so studies are needed for future generalisation of the results to other wider populations of COPD patients, in particular those treated in primary care settings. Another limitation to be taken into account is that our model considered as an outcome the clinical control of COPD estimated and reported by the specialist during the medical visit. This estimate is based on compliance with treatment goals according to good clinical practice guidelines [1,2,11,31]. This introduces variability, as there is no universal standard, no standardised method to measure control objectively. In addition, although this study included a relatively large sample size, the number of outcomes was limited, which may increase the risk of overfitting the model. Another aspect to consider is the retrospective nature of the study, so its analysis introduces the possibility that unrecognised factors, inaccurate measurements or missing data may lead to measurement biases that may influence the results, even though the recorded data were checked by data managers and members of the EPOCONSUL Audit Committee. Therefore, future prospective research is needed to evaluate this quantitative score as a predictor of COPD outcomes.

## 5. Conclusions

In this study, we developed and validated an easy-to-use scoring system that quantifies the criteria defining the degree of clinical control of COPD. This system will aid in measuring COPD control and its progression during patient follow-up. Further studies are required to prospectively evaluate our scoring system as a predictor of outcomes in COPD across different settings and to elucidate its clinical utility.

## Figures and Tables

**Table 1:** Characteristics of the development and validation cohorts.

	Development Cohort (n = 485)	Validation Cohort (n = 341)
Demographic and clinical characteristics		
Gender (male), n (%)	341 (70.3)	240 (70.4)
Age (years), m (SD)	68.9 (9.4)	69.4 (8.8)
Tobacco Current smokers, n (%) Ex-smokers, n (%) Pack-years, m (SD)	142 (29.3) 343 (70.7) 47.9 (22.7)	81 (23.8) 260 (76.2) 47.7 (23.0)
BMI kg/m^2^, m (SD)	27.3 (5.4)	27.1 (4.4)
Charlson index, median, IQR Charlson index ≥ 3, n (%)	1 (1–3) 139 (28.7)	1 (1–3) 92 (27.1)
Dyspnoea (MRC-m) ≥ 2, n (%)	278 (57.3)	192 (56.3)
CAT questionnaire > 10, n (%)	133 (70.4)	107 (78.1)
Chronic bronchitis criteria, n (%)	217 (44.7)	153 (44.9)
Post-FEV_1_, % predicted, m (SD)	52.7 (17.6)	52.8 (18.8)
KCO, % predicted, m (SD)	67.2 (21.4)	66.3 (22.6)
Had severe exacerbations in previous year, n (%)	133 (27.4)	89 (26.1)
BODE value, median, IQR	4 (2–5)	4 (2–5)
GOLD group, n (%) A B C D	75 (29.6) 68 (26.9) 21 (8.3) 89 (35.2)	55 (29.3) 35 (26.1) 20 (10.6) 59 (31.4)
GesEPOC risk level, n (%) Low risk level High risk level	166 (39.8) 251 (60.2)	111 (38.1) 180 (61.9)
Inhaled therapy, n (%) LAMA LABA LAMA-LABA combination LABA+ ICS combination Triple therapy	27 (5.6) 2 (0.4) 182 (37.6) 37 (7.6) 236 (48.8)	25 (7.4) 2 (0.6) 131 (38.9) 23 (6.8) 156 (46.3)
Degree of clinical control reported at the visit by the attending doctor ^#^ Uncontrolled patient, n (%) Controlled patient, n (%)	155 (32) 330 (68.0)	110 (32.3) 231 (67.7)
Long-term oxygen therapy, n (%)	120 (24.7)	88 (25.8)
Home ventilation, n (%)	53 (10.9)	28 (8.2)
Respiratory rehabilitation, n (%)	81 (16.7)	57 (16.7)
**Resources in care**		
Level of complexity of hospital Tertiary, n (%) Secondary, n (%)	348 (71.8) 137 (28.2)	243 (71.3) 98 (28.7)
University Hospital, n (%)	337 (69.5)	211 (72.8)
Attended in specialized COPD outpatient clinic, n (%)	192 (39.8)	117 (34.3)
Scheduled follow-up visits, n (%) <6 months 6–12 months >12 months	210 (46.1) 146 (43.8) 48 (10.5)	151 (45.3) 198 (43.4) 36 (10.8)

Data presented as mean (SD) or number (percentage) or median (interquartile range); BMI: body mass index; CAT: COPD Assessment Test; FEV_1_%: post-bronchodilator FEV_1_ percent predicted; KCO%: percent predicted carbon monoxide diffusion factor BODE: body mass index, airflow obstruction, dyspnoea, and exercise capacity; GOLD: Global Initiative for Chronic Obstructive Lung Disease; GesEPOC: Spanish National Guideline for COPD; LABA: long-acting beta-2 agonists; LAMA: long-acting antimuscarinic agents; CSI: Inhaled corticosteroids; Triple therapy: LAMA+LABA+CSI; Hospital complexity level II (secondary hospital): from 5 to 10 medical specialties; capacity of 200–800 beds; often referred to as provincial hospitals. Hospital complexity level III (tertiary hospital): Highly specialized equipment and staff, capacity of 300–1500 beds. ^#^ degree of COPD control estimated and reported by the physician at the time of visit.

**Table 2:** Criteria to define the degree of clinical control validated and established in GesEPOC in the development cohort.

COPD Clinical Control Grade Estimated and Reported by the Physician at the Visit (N = 485)	Patients with Controlled COPD	Patients with Poorly Controlled COPD	*p* Value	Unadjusted OR	Unadjusted OR (95% CI)
Degree of dyspnoea adjusted for obstruction severity *					
Yes	88 (46.1)	103 (53.9)	<0.001	5.447	(3.604–8.234)
Not	242 (82.3)	52 (17.7)		1	1
Use rescue inhaler more than three times per week					
Yes	41 (27.7)	107 (72.3)	<0.001	15.713	(9.800–25.194)
Not	289 (85.8)	48 (14.2)		1	1
Walk less than 30 min a day					
Yes	141 (54.7)	117 (45.3)	<0.001	4.127	(2.695–6.319)
Not	189 (83.3)	38 (16.7)		1	1
Sputum colour is purulent (with colour)					
Yes	2 (66.7)	1 (33.3)	0.959	1.065	(0.096–11.384)
Not	328 (68)	154 (32)		1	1
Exacerbations of COPD in the last 3 months					
Yes	54 (34.6)	102 (65.4)	<0.001	9.836	(6.324–15.301)
Not	276 (83.9)	53 (16.1)		1	1

Data are represented as absolute (relative) frequencies. * dyspnoea (MRC-m) > 1 if Post-FEV1 ≥50% predicted or dyspnoea (MRC-m) > 2 if Post-FEV1 < 50% predicted.

**Table 3:** Multivariable Logistic Model for the non-control of COPD in the development cohort.

Variable	β Coefficient (SE)	*p* Value	Adjusted OR	(CI 95%)	Scoring of Degree Clinical Control of COPD
Degree of dyspnoea adjusted for obstruction severity *	1.367	<0.001	3.922	(2.289–6.719)	2
Use rescue inhaler more than three times per week	2.045	<0.001	7.726	(4.517–13.212)	3
Walk less than 30 min a day	0.708	<0.012	2.030	(1.165–3.536)	1
Exacerbations of COPD in the last 3 months	1.722	<0.001	5.598	(3.271–9.579)	2

* dyspnoea (MRC-m) > 1 if Post-FEV1 ≥50% predicted or dyspnoea (MRC-m) > 2 if Post-FEV1 < 50% predicted.

## Data Availability

Dataset available on request from the authors.

## Supplementary Materials

The following supporting information can be downloaded at: https://www.mdpi.com/article/10.3390/jcm14030707/s1, Table S1: TRIPOD Checklist: Prediction Model Development; Table S2: The inclusion criteria and exclusion criteria; Table S3: Hospital-related and patient-related variables; Table S4: Participants in 2021 EPOCONSUL study; Table S5: Clinical control of COPD according to GesEPOC criteria; Table S6: Characteristics of patients included and excluded in this analysis.

## Abbreviations

The following abbreviations are used in this manuscript:

COPD	Chronic Obstructive Pulmonary Disease
AUC	Area Under Curve
GesEPOC	Spanish COPD Guide

## References

[1] Miravitlles M., Calle M., Molina J., Almagro P., Gómez J.-T., Trigueros J.A., Cosío B.G., Casanova C., López-Campos J.L., Riesco J.A. (2022). Spanish COPD guidelines (GesEPOC) 2021: Updated pharmacological treatment of stable COPD. Arch. Bronconeumol..

[2] Agustí A., Celli B.R., Criner G.J., Halpin D., Anzueto A., Barnes P., Bourbeau J., Han M.K., Martinez F.J., de Oca M.M. (2023). Global initiative for chronic obstructive lung disease 2023 report: GOLD executive summary. Arch. Bronconeumol..

[3] Soler-Cataluña J.J., Alcázar-Navarrete B., Miravitlles M. (2014). The concept of control in COPD: A new proposal for optimising therapy. Eur. Respir. J..

[4] Soler-Cataluña J.J., Marzo M., Catalán P., Miralles C., Alcazar B., Miravitlles M. (2018). Validation of clinical control in COPD as a new tool for optimizing treatment. Int. J. Chron. Obstruct. Pulmon. Dis..

[5] Tashiro H., Takahashi K. (2023). Clinical Impacts of interventions for physical activity and sedentary behavior on patients with chronic obstructive pulmonary disease. J. Clin. Med..

[6] Gunen H., Kokturk N., Naycı S., Ozkaya S., Yıldız B.P., Turan O., Gumus A., Akgun M., Gurgun A., Ogus C. (2022). The CO-MIND Study: Chronic obstructive pulmonary disease management in daily practice and its implications for improved outcomes according to GOLD 2019 perspective. Int. J. Chron. Obstruct. Pulmon. Dis..

[7] Vanfleteren L.E.G.W., Lindberg A., Zhou C., Nyberg F., Stridsman C. (2023). Exacerbation risk and mortality in global initiative for chronic obstructive lung disease group A and B patients with and without exacerbation history. Am. J. Respir. Crit. Care. Med..

[8] Barrecheguren M., Kostikas K., Mezzi K., Shen S., Alcazar B., Soler-Cataluña J.J., Miravitlles M., Wedzicha J.A. (2020). COPD clinical control as a predictor of future exacerbations: Concept validation in the SPARK study population. Thorax..

[9] Price D., West D., Brusselle G., Gruffydd-Jones K., Jones R., Miravitlles M., Rossi A., Hutton C., Ashton V.L., Stewart R. (2014). Management of COPD in the UK primary-care setting: An analysis of real-life prescribing patterns. Int. J. Chron. Obstruct. Pulmon. Dis..

[10] Calle Rubio M., Rodríguez Hermosa J.L., de Torres J.P., Marín J.M., Martínez-González C., Fuster A., Cosío B.G., Peces-Barba G., Solanes I., Feu-Collado N. (2021). COPD Clinical Control: Predictors and long-term follow-up of the CHAIN cohort. Respir. Res..

[11] Calle Rubio M., López-Campos J.L., Soler-Cataluña J.J., Alcázar Navarrete B., Soriano J.B., Rodríguez González-Moro J.M., Ferrer M.E.F., Hermosa J.L.R. (2017). EPOCONSUL Study. Variability in adherence to clinical practice guidelines and recommendations in COPD outpatients: A multi-level, cross-sectional analysis of the EPOCONSUL study. Respir. Res..

[12] Moons K.G., Altman D.G., Reitsma J.B., Ioannidis J.P., Macaskill P., Steyerberg E.W., Vickers A.J., Ransohoff D.F., Collins G.S. (2015). Transparent reporting of a multivariable prediction model for individual prognosis or diagnosis (TRIPOD): Explanation and elaboration. Ann. Intern. Med..

[13] Agustí A., Edwards L.D., Rennard S.I., MacNee W., Tal-Singer R., Miller B.E., Vestbo J., Lomas D.A., Wouters E., Crim C. (2012). Persistent systemic inflam mation is associated with poor clinical outcomes in COPD: A novel phenotype. PLoS ONE.

[14] Singh D., Agusti A., Anzueto A., Barnes P.J., Bourbeau J., Celli B.R., Criner G.J., Frith P., Halpin D.M.G., Han M. (2019). Global strategy for the diagnosis, management, and prevention of chronic obstructive lung disease: The GOLD science committee report 2019. Eur. Respir. J..

[15] Bestall J.C., Paul E.A., Garrod R., Garnham R., Jones P.W., Wedzicha J.A. (1999). Usefulness of the medical research council (MRC) dyspnoea scale as a measure of disability in patients with chronic obstructive pulmonary disease. Thorax.

[16] Ramon M.A., Esquinas C., Barrecheguren M., Pleguezuelos E., Molina J., Quintano J.A., Roman-Rodríguez M., Naberan K., Llor C., Roncero C. (2017). Self-reported daily walking time in COPD: Relationship with relevant clinical and functional characteristics. Int. J. Chron. Obstruct. Pulmon. Dis..

[17] Nattino G., Finazzi S., Bertolini G.A. (2016). New test and graphical tool to assess the goodness of fit of logistic regression models. Stat. Med..

[18] Halpin D.M.G., de Jong H.J.I., Carter V., Skinner D., Price D. (2019). Distribution, temporal stability and appropriateness of therapy of patients with COPD in the UK in relation to GOLD 2019. eClinicalMedicine.

[19] Albitar H.A.H., Iyer V.N. (2020). Adherence to global initiative for chronic obstructive lung disease guidelines in the real world: Current understanding, barriers, and solutions. Curr. Opin. Pulm. Med..

[20] Singh D., Holmes S., Adams C., Bafadhel M., Hurst J.R. (2021). Overcoming therapeutic inertia to reduce the risk of COPD exacerbations: Four action points for healthcare professionals. Int. J. Chron. Obstruct. Pulmon. Dis..

[21] Fiore M., Ricci M., Rosso A., Flacco M.E., Manzoli L. (2023). Chronic obstructive pulmonary disease overdiagnosis and overtreatment: A meta-analysis. J. Clin. Med..

[22] Tzouvelekis A., Kyriakopoulos C., Gerogianni I., Rapti A., Michailidis V., Dimoulis A., Steiropoulos P., Styliara P., Kostikas K., Gogali A. (2024). Real world study on the reasons for escalation or de-escalation of inhaled therapies in COPD patients: The STEPINCOPD multicenter observational study. J. Chronic Obstr. Pulm. Dis..

[23] Woodruff P.G., Agusti A., Roche N., Singh D., Martinez F.J. (2015). Current concepts in targeting chronic obstructive pulmonary disease pharmacotherapy: Making progress towards personalised management. Lancet.

[24] Moretta P., Cavallo N.D., Candia C., Lanzillo A., Marcuccio G., Santangelo G., Marcuccio L., Ambrosino P., Maniscalco M. (2024). Psychiatric Disorders in patients with chronic obstructive pulmonary disease: Clinical significance and treatment strategies. J. Clin. Med..

[25] Jenkins C.R., Postma D.S., Anzueto A.R., Make B.J., Peterson S., Eriksson G., Calverley P.M. (2015). Reliever salbutamol use as a measure of exacerbation risk in chronic obstructive pulmonary disease. BMC Pulmon. Med..

[26] Garcia-Aymerich J., Lange P., Benet M., Schnohr P., Antó J.M. (2007). Regular physical activity modifies smoking-related lung function decline and reduces risk of chronic obstructive pulmonary disease: A population-based cohort study. Am. J. Respir. Crit. Care Med..

[27] Kim T., Kim H., Shin S.H., Im Y., Kong S., Choi H.S., Zo S., Kim S.H., Choi Y., Kang D. (2024). Association of moderate-to-vigorous physical activity with reduction of acute exacerbation in COPD patients using a dual ultra-long-acting bronchodilators. Sci. Rep..

[28] Nici L., Mammen M.J., Charbek E., Alexander P.E., Au D.H., Boyd C.M., Criner G.J., Donaldson G.C., Dreher M., Fan V.S. (2020). Pharmacologic management of chronic obstructive pulmonary disease. An official american thoracic society clinical practice guideline. Am. J. Respir. Crit. Care Med..

[29] Nathan R.A., Sorkness C.A., Kosinski M., Schatz M., Li J.T., Marcus P., Murray J.J., Pendergraft T.B. (2004). Development of the asthma control test: A survey for assessing asthma control. J. Allergy Clin. Immunol..

[30] Ye L., Gao X., Tu C., Du C., Gu W., Hang J., Zhao L., Jie Z., Li H., Lu Y. (2021). Comparative analysis of effectiveness of asthma control test-guided treatment versus usual care in patients with asthma from China. Respir. Med..

[31] Mannino D., Siddall J., Small M., Haq A., Stiegler M., Bogart M. (2022). Treatment patterns for chronic obstructive pulmonary disease (COPD) in the United States: Results from an observational cross-sectional physician and patient survey. Int. J. Chron. Obstruct. Pulmon. Dis..

