# Supplementary material for: Development and Validation of a Quantitative Score for the Criteria Clinical Control in Stable COPD Proposed in the Spanish COPD Guidelines (GesEPOC): Results of the EPOCONSUL Audit
Source: J Clin Med. 2025 Jan 22;14(3):707. doi: 10.3390/jcm14030707 (PMC11818294; doi:10.3390/jcm14030707)
Supplement: Supplementary file 1 [file jcm-14-00707-s001.zip › jcm-3417556-supplementary.pdf]

SUPPLEMENTARY

Table S1. TRIPOD Checklist: Prediction Model Development

**Section/Topic**	**ı**	**Checklist Item**	**Page**
**Title and abstract**			
Title	1	Identify the study as developing and/or validating a multivariable prediction model, the target population, and the outcome to be predicted.	1
Abstract	2	Provide a summary of objectives, study design, setting, participants, sample size, predictors, outcome, statistical analysis, results, and conclusions.	1
**Introduction**			
Background and objectives	3a	Explain the medical context (including whether diagnostic or prognostic) and rationale for developing or validating the multivariable prediction model, including references to existing models.	2
	3b	Specify the objectives, including whether the study describes the development or validation of the model or both.	2
**Methods**			
Source of data	4a	Describe the study design or source of data (e.g., randomized trial, cohort, or registry data), separately for the development and validation data sets, if applicable.	3
	4b	Specify the key study dates, including start of accrual; end of accrual; and, if applicable, end of follow-up.	NA
Participants	5a	Specify key elements of the study setting (e.g., primary care, secondary care, general population) including number and location of centres.	3-4 Table S3
	5b	Describe eligibility criteria for participants.	Table S1
	5c	Give details of treatments received, if relevant.	NA
Outcome	6a	Clearly define the outcome that is predicted by the prediction model, including how and when assessed.	4
	6b	Report any actions to blind assessment of the outcome to be predicted.	NA
Predictors	7a	Clearly define all predictors used in developing or validating the multivariable prediction model, including how and when they were measured.	4, Table S4
	7b	Report any actions to blind assessment of predictors for the outcome and other predictors.	NA
Sample size	8	Explain how the study size was arrived at.	NA
Missing data	9	Describe how missing data were handled (e.g., complete-case analysis, single imputation, multiple imputation) with details of any imputation method.	4,5
Statistical analysis methods	10a	Describe how predictors were handled in the analyses.	NA
	10b	Specify type of model, all model-building procedures (including any predictor selection), and method for internal validation.	5
	10d	Specify all measures used to assess model performance and, if relevant, to compare multiple models.	5
Risk groups	11	Provide details on how risk groups were created, if done.	NA
**Results**			
Participants	13a	Describe the flow of participants through the study, including the number of participants with and without the outcome and, if applicable, a summary of the follow-up time. A diagram may be helpful.	5
	13b	Describe the characteristics of the participants (basic demographics, clinical features, available predictors), including the number of participants with missing data for predictors and outcome.	6-7, Table
Model development	14a	Specify the number of participants and outcome events in each analysis.	7-8
	14b	If done, report the unadjusted association between each candidate predictor and outcome.	7-8

Model specification	15a	Present the full prediction model to allow predictions for individuals (i.e., all regression coefficients, and model intercept or baseline survival at a given time point).	8-9
	15b	Explain how to the use the prediction model.	4, 9-10
Model performance	16	Report performance measures (with CIs) for the prediction model.	8-10
**Discussion**			
Limitations	18	Discuss any limitations of the study (such as nonrepresentative sample, few events per predictor, missing data).	12
Interpretation	19b	Give an overall interpretation of the results, considering objectives, limitations, and results from similar studies, and other relevant evidence.	11-12
Implications	20	Discuss the potential clinical use of the model and implications for future research.	11-12
**Other information**			
Supplementary information	21	Provide information about the availability of supplementary resources, such as study protocol, Web calculator, and data sets.	12
Funding	22	Give the source of funding and the role of the funders for the present study.	12

NA, non-applicable

Table S2. The inclusion criteria and exclusion criteria

The inclusion criteria	- patients aged ≥40 years<br>- smokers or ex-smokers (of at least 10 pack-years)<br>- COPD diagnosed on the basis of spirometric tests (FEV1/FVC post-bronchodilation<0.7 or FEV1/FVC pre-bronchodilation<0.7 and FEV1≥80%, if there is no bronchodilation reversibility testing available
The exclusion criteria	- lack of follow-up for at least 1 year in a respiratory outpatient clinic<br>- participating in a clinical trial

Table S3. Hospital-related and patient-related variables

Hospital-related variables

1. Autonomous Community of Spain
2. Province where the hospital belongs
3. Population assigned for admission to the hospital
5. Level of hospital: primary-level I: has few specialities, mainly internal medicine, obstetrics-gynecology, pediatrics, and general surgery; limited laboratory services are available for general but not for specialized pathological analysis; bed capacity ranges from 30-200 beds. Secondary-level II: highly differentiated by function with five to ten clinical specialities and bed capacity from 200-800 beds; often referred to as provincial hospital. Tertiary-level III: highly specialized staff and technical equipmen, ICU and specialized imaging units; clinical services are highly differentiated by function; bed capacity from 300-1500 beds; often referred to as central, regional or tertiary-level hospital.Is it a university hospital?
6. Is there inpatient clinic in the Respiratory unit?
7. Respiratory hospitalization beds
8. Are there Pulmonology residents in the hospital?
9. Total number of Pulmonology residents in the beginning of the study
10. Number of staff pulmonologists in the Unit (excluding residents)
11. Which of the following specialized respiratory outpatient clinics are available in your Respiratory unit?
12. How many minutes do you usually have for a scheduled first time visit in a specialized respiratory outpatient clinic?
13. How many minutes do you usually have for a scheduled follow-up visit in a specialized respiratory outpatient clinic?
14. How many minutes do you usually have for a scheduled first-time visit in the general respiratory outpatient clinic?
15. How many minutes do you usually have for a scheduled follow-up visit in the general respiratory outpatient clinic?
16. Is there a nursing respiratory in the outpatient clinic?
17. How many nursing respiratory outpatient clinics are there?
18. Does your Respiratory unit have a specialized COPD respiratory outpatient clinic?
19. How many scheduled visits are there in a week in the specialized COPD respiratory outpatient clinic?
20. Does the specialized COPD respiratory outpatient clinic have a nurse available?
21. What is the nurse profile?
22. Does the Unit have an organized educational program on inhaler use for the outpatient COPD patients?
23. Who runs it?
24. Does the center have forced spirometry available?
25. Does the center have static lung volumes measurement available?
26. Does the center have diffusing capacity measurement available?
27. Does the center have respiratory muscle strength measurement available?
29. Does the center have sputum eosinophil count available?
30. Indicate how often you use it in your clinical practice
31. Does the center have sputum culture available?

32. Does the center have cardiorespiratory exercise testing available?
33. Does the center have echocardiography available?
34. Does the center have simple thoracic X-ray available?
35. Does the center have chest CT scan available?
36. Does the center have total serum IgE determination available?
37. Does the center have serum Alfa-1-antitrypsin determination available?
38. Does the center have genetic testing for Alfa-1-antitrypsin deficiency available?
39. Does the center have 6MWT available?
40.
41. Does the center have CAT questionnaire available in the outpatient clinic?
42. Does the center have a respiratory rehabilitation program available?
43. Of what type:
46. Is there any type of protocoled nutritional support for COPD patients?

Patient-related variables

1. Investigator
2. Center
3. Follow-up start and final date
4. Visit type
5. Patient gender
6. Patient age
7. Years of clinical follow-up
8. Smoking history available
9. Number of cigarettes per day
10. Number of years smoking
11. Last spirometry testing
12. Post-bronchodilation spirometry - Date
13. Post-bronchodilation spirometry - FEV1 (ml)
14. Post-bronchodilation spirometry - FEV1 (%)
15. Post-bronchodilation spirometry - FVC (ml)
16. Post-bronchodilation spirometry - FVC (%)
17. Post-bronchodilation spirometry - FEV1/FVC
18. Does the patient participate in any clinical trials or research projects?
19. Does the patient meet all the inclusion criteria?
20. What Unit referred the patient to the Respiratory outpatient clinic?
21. What is the type of outpatient clinic that follows the case?
22. Date of current visit
23. Date of previous scheduled follow-up visit
24. What is the smoker status of the patient?
25. Patient's weight in the last spirometry report (Kg)
26. Patient's height in the last spirometry report(m)
27. Are the comorbidities defined in the clinical record, including?
28. Cardiopathy
29. Peripheral arterial disease
30. Arterial hypertension
31. Dyslipidemia
32. Neurologic disease
33. Sleep apnea

34. Depression
35. Anxiety
36. Chronic respiratory disease
37. Of what type:
38. Other respiratory diseases
39. Asthma
40. Rhinitis
41. Digestive disorders
42. Metabolic syndrome
43. Diabetes
44. Chronic renal disease
45. Neoplastic disease
46. AIDS
47. Connective tissue diseases
48. Bone, joint and muscle disorders
49. Total Charlson index
50. Bronchodilator reversibility testing
51. % reversibility
52. Is serum Alfa-1-antitrypsin determination available?
53. Measured value
54. Chronic bronchitis criteria
55. Sputum color is purulent
56. Is sputum culture performed on any occasion?
57. Symptoms suggestive of asthma,
58. Walk less than 30 minutes (on average) per day
59. Cardiopulmonary exercise testing performed on any occasion?
60. Is Chest CT scan carried out on any occasion?
61. Reason for performing chest CT scan?
62. Is 6MWT performed on any occasion?
63. Date of 6MWT
64. Distance recorded
65. Are Symptoms or quality of life questionnaires determined on any occasion?
66. Date CAT
67. Score CAT
68. Is Lung volume measurement performed on any occasion?
69. Is diffusion capacity performed on any occasion?
70. Are Arterial blood gases performed on any occasion?
71. BODE index calculated on any occasion?
72. Value BODE
73. Is the BODEx index calculated on any occasion?
74. Value BODEx
75. Dyspnea grade according to mMRC
76. Does the patient currently meet the clinical criteria for chronic cough and/or chronic expectoration?
77. Is there chronic colonization by any microorganism?
78. Is the number of moderate/severe exacerbations in the last 12 months recorded during the visit?

79. Number
80. Review clinical record and indicate number
81. Is the number of hospital admissions in the last 12 months recorded?
82. Number
83. Review clinical record and indicate number
84. Exacerbations in the last 3 months
85. Is smoking history recorded during the visit?
86. Is exercise routine recorded during the visit?
87. Is annual influenza vaccination recorded during the visit?
88. Is pneumococcal vaccination recorded during the visit?
89. Specify if any complementary examination is considered during the current visit
90. Bronchodilation reversibility testing
91. Lung volumes
92. Diffusing capacity
93. Arterial blood gases
94. 6MWT
95. Total serum IgE
96. ReasonEchocardiography
97. CT
98. Symptoms questionnaires
99. Is the current COPD medication reported in the clinical record?
100. Which of the following pharmacologic treatments is the patient prescribed:
101. Is treatment adherence evaluated in some way?
102. Is the grade of satisfaction with the inhaler devise recorded?
103. Is inhalation technique evaluated in some way?
104. Use rescue inhaler per week
105. Was the pharmacologic treatment modified compared with the previously used?
106. Indicate any changes
107. Long-term oxygen therapy
108. Home mechanical ventilation
109. Does the patient participate in a respiratory rehabilitation program?
110. Is a specific intervention for smoking cessation offered?
111. Indicate
112. Is exercise recommended during the visit?
113. Is vaccination recommended during the visit?
114. Indicate
115. Is COPD severity defined in the clinical record?
116. Indicate
117. Is the GesEPOC phenotype defined in the clinical record?
118. Indicate
119. Is the next scheduled visit indicated in the clinical record?
120. Next follow-up visit in. (months)
121. Was the patient discharged from the clinic?

Table S4. Participants in 2021 EPOCONSUL study

Andalucía: José Calvo Bonachera. H. Torrecárdenas. Almeria, Virginia Almadana Pacheco. H. U Virgen de la Macarena. Malaga, Francisco Marin Sanchez. H. U. Virgen de la Victoria. Malaga, J. L Lopez Campos. H. U Virgen del Rocio. Sevilla.

Aragón: Maria Angeles Gotor Lazaro. Hospital Universitario Miguel Servet. Ana Boldova. Zaragoza, Hospital Royo Villanova. Zaragoza, Virginia Mo, Cristina Aguilar Paesa. H. Clinico. Zaragoza.

Asturias: Marta Iscar Urrutia, Ana Pando Sandoval, Cristina Hernández González. Hospital Universitario Central de Asturias. Oviedo,

Murcia: Maria Jesús Avilés Inglés. Hospital General. Universitario Reina Sofía, Juan Miguel Sánchez Nieto. Hospital Morales Meseguer, Mª Carmen Fernández Sánchez. H. Universitario Rafael Méndez. Murcia.

Canarias: Marco Acosta Sorense. Hospital Universitario Nuestra Señora de la Candelaria. Tenerife.

Cantabria: Beatriz Abascal. H. U de Valdecilla. Santander.

Castilla y la Mancha: José Alfonso García Guerra. H. Mancha Centro. Alcazar de San Juan. Ciudad Real.

Castilla y León: Ana Pueyo. Hospital Universitario de Burgos, José Luis Fernández Sanchez, Maria Bartol Sanchez, Tamara Clavero Sanchez, Laura Gil Pintor. H. U. de Salamanca.

Cataluña: Miriam Barrecheguren. H Val de Hebron. Barcelona, Noelia Pablos Mateos. H. Sant Joan de Déu de Martorell. Barcelona, Sandra Marin. Hospital Dos de Maig de Barcelona, Annie Navarro. Hospital U Mútua de Terrassa. Barcelona, Elena de Miguel Campos. Hospital Moisés Broggi Sant Joan Despí. Barcelona.

Valencia: Jose Maria Tordera. Hospital Universitario La Fe de Valencia, Dolores Martinez Pitarch. Hospital LLuis Alcanys de Xátiva. Valencia, Marta Palop Cervera. Hospital de Sagunto. Valencia, Lia Alonso Tomás, Marta Solé Delgado. H Arnau de Vilanova. Valencia, Cruz Gonzalez Villaescusa. Hospital Clínico Universitario de Valencia, Eusebi Chiner Vives. H. U. San Juan. Alicante.

Extremadura: Francisca Lourdes Marquez, Luis Miguel Sierra Murillo. Hospital Universitario de Badajoz, Juan Antonio Riesco. H. San Pedro de Alcantara. Mirian Torres González. Hospital Virgen del Puerto de Plasencia,

Baleares: Francisco Fanjul Losa. Hospital Universitario Son Espases. Palma de Mallorca, Antonia Fuster Gomila. Hospital Universitario Son Lltzer. Palma de Mallorca.

Madrid: Soledad Alonso Viteri. H de Torrejon. Madrid, Aurora Solier. H Ramon y Cajal. Madrid, Andrea Yordi. H. Infanta Elena. Valdemoro. Madrid, Nuria Arenas, Blas Rojo. Hospital Infanta Sofía San Sebastián de los Reyes. Madrid, Juan Luis Rodriguez

Hermosa, Gianna Vargas Centanaro. H. Clinico San Carlos. Madrid, Manuel Valle Falcones. H. U Puerta de Hierro Majadahonda. Madrid, Tamara Alonso Perez, Rosa Mar Gómez Punter, Elena García Castillo. H. La Princesa. Madrid, J. De Miguel, Zichen Ji. H. U. Gregorio Marañon. Madrid, Carolina Maria Gotera Rivera. Fundación Jimenez Diaz. Madrid.

Madrid, Carolina Maria Gotera Rivera. Fundación Jimenez Diaz. Madrid.

Navarra: Jose Espinoza Perez. Complejo Hospitalario de Navarra.

País Vasco: Maria Milagros Iriberri Pascual, Patricia Sobradillo Ecenarro. H de Cruces. Vizcaya, Raquel Sánchez Juez. Hospital Universitario de Basurto. Vizcaya, Cristobal Esteban Gonzalez. Hospital de Galdakao. Vizcaya.

Table S5. Clinical control of COPD according to GesEPOC criteria

**Criteria for the level of clinical control of COPD**		
**Low clinical impact** (at least 3 of the 4 criteria must be met)		
	FEV_1 ≥50%	FEV_1 <50%
- What is the color of the sputum of the last few days?	White / clean or without sputum	White / clean or without sputum
- Dyspnea	0-1	0-2
- How many times did you use rescue medication in the last week?	< 3 times / week	< 3 times / week
- How much time (on average) have you walked per day in the last week?	≥ 30 minutes per day	≥ 30 minutes per day
**Clinical stability** (criteria must be met)		
- Exacerbations in the last 3 months	None	
**Good clinical control of COPD**	Low impact + stability	

Criteria of poor clinical control of COPD: rescue medication assessed at the visit, use rescue inhaler more than three times per week; the degree of dyspnea (MRC-m), >1 if post-FEV_1 ≥50% predicted or >2 if post-FEV_1 <50% predicted; self-reported physical activity referred at the visit , walk less than 30 minutes per day; exacerbations of COPD in the last 3 months, one or more that required the use of systemic corticosteroids and/or a course of antibiotics.

Table S6. Characteristics of patients included and excluded in this analysis

	Patients included (n=826)	Patients excluded (n= 3399)	p
**Demographic and clinical characteristics**			
Gender (male), n (%)	581 (70.3)	2492 (73.4)	0.080
Age (years), m (SD)	69.1 (9.1)	70.2 (9.3)	0.004
Current smokers, n (%)	223 (27)	830 (24.4)	0.124
BMI kg/m2, m (SD)	27.2 (5.0)	27.9 (5.7)	0.001
Charlson index ≥3, n (%)	231 (28)	954 (28.1)	0.947
Dyspnea (MRC-m) ≥2, n (%)	470 (56.9)	1317 (55.7)	0.561
Post-FEV_1, % predicted, m (SD)	52.7 (18.1)	53.2 (18.6)	0.527
Number of moderate exacerbations in the last year, median, IQR	1 (0 – 1)	0 (0 - 1)	0.001
Number of severe exacerbations in previous year, median, IQR	0 (0 – 1)	0 (0 – 0)	0.001
BODEx value, median, IQR	3 (1-5)	3 (1-5)	0.386
GOLD group, n (%)			0.413
• A	130 (29.5)	329 (28.1)	
• B	122 (27.7)	326 (27.8)	
• C	41 (9.3)	143 (12.2)	
• D	148 (33.6)	374 (31.9)	
Uncontrolled patient according to GesEPOC criteria, (%)	410 (49.6)	475 (48.6)	0.651
Long-term oxygen therapy, n (%)	208 (25.2)	851 (25)	0.931
Home ventilation, n (%)	81 (9.8)	281 (8.3)	0.156
Attended in specialized COPD outpatient clinic, n (%)	309 (37.5)	1311 (38.7)	0.527
